# Strengthening the Structural Behavior of Web Openings in RC Deep Beam Using CFRP

**DOI:** 10.3390/ma13122804

**Published:** 2020-06-22

**Authors:** Nurul Izzati Rahim, Bashar S. Mohammed, Amin Al-Fakih, M. M. A. Wahab, M. S. Liew, Abdullah Anwar, Y. H. Mugahed Amran

**Affiliations:** 1 Civil and Environmental Engineering Department, Universiti Teknologi PETRONAS, Bandar Seri Iskandar 32610, Malaysia; nurul_18001893@utp.edu.my (N.I.R.); mubarakwahab@utp.edu.my (M.M.A.W.); shahir_liew@utp.edu.my (M.S.L.); abdullah_18001789@utp.edu.my (A.A.); 2 Department of Civil Engineering, College of Engineering, Prince Sattam Bin Abdulaziz University, Alkharj 11942, Saudi Arabia; m.amran@psau.edu.sa; 3 Department of Civil Engineering, Faculty of Engineering and IT, Amran University, Quhal 9677, Amran, Yemen

**Keywords:** RC deep beams, web opening, CFRP, shear behavior, strengthening

## Abstract

Deep beams are more susceptible to shear failure, and therefore reparation is a crucial for structural reinforcements. Shear failure is structural concrete failure in nature. It generally occurs without warning; however, it is acceptable for the beam to fail in bending but not in shear. The experimental study presented the structural behavior of the deep beams of reinforced concrete (RC) that reinforces the web openings with externally connected carbon fiber reinforced polymer (CFRP) composite in the shear zone. The structural behavior includes a failure mode, and cracking pattern, load deflection responses, stress concentration and the reinforcement factor were investigated. A total of nine reinforced concrete deep beams with openings strengthened with CFRP and one control beam without an opening have been cast and tested under static four-point bending load till failure. The experimental results showed that the increase the size of the opening causes an increase in the shear strength reduction by up to 30%. Therefore, the larger the openings, the lower the capability of load carriage, in addition to an increase in the number of CFRP layers that could enhance the load carrying capacity. Consequently, utilization of the CFRP layer wrapping technique strengthened the shear behavior of the reinforced concrete deep beams from about 10% to 40%. It was concluded that the most effective number of CFRP layers for the deep beam with opening sizes of 150 mm and 200 mm were two layers and three layers, respectively.

## 1. Introduction

**Figure 1:**
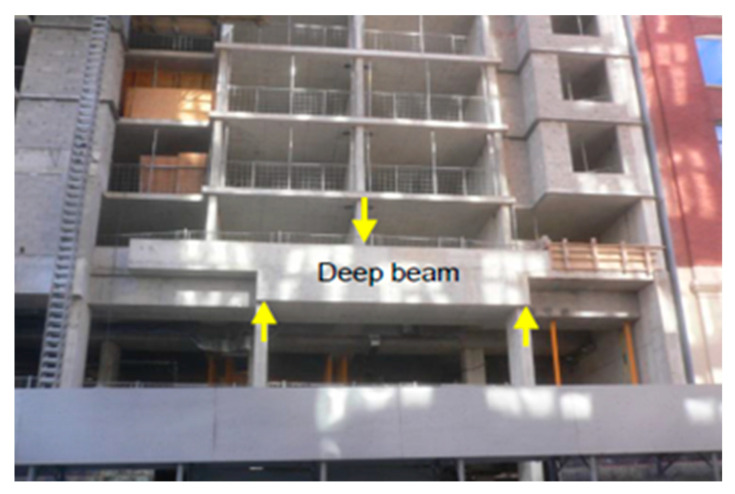
Deep Beam in a multi-story building [2].

Reinforced Concrete (RC) deep beams are the most common structural members in modern construction, especially in high rise buildings. It is commonly used as load distribution elements, such as transfer girders in high rise buildings, bent caps in bridges and pile caps in foundations [1,2] as shown in Figure 1.

Deep beams of RC transfer the load as a simply supported beam to the support through a compression mechanism [3,4]. The beam which possesses greater height (*h*) as compared to its length of span (*l*) is referred to as a deep beam. The length to height ratio (*l/h*) should be less than a certain value with respect to the deep beam [5]. According to the American Concrete Institute (ACI) 318-14 [3], the deep beam is defined in two aspects. Firstly, the beam having a clear span to depth ratio of less than or equal to four times the beam depth. Secondly, the area of the concentrated load lies within twice the member depth from the face of support [3,6]. The deep beam is considered a complex phenomenon, as various experimental research has been conducted previously, but its structural behavior is still unreliable [7]. Most of the researchers concluded that failure is greatly influenced by its shear capacity. Thus, making shear behavior one of the key factors in analyzing the structural behavior and safety [7]. Deep beam comprises non-linear deformation strains and non-flexural behavior [7,8]. The elastic theory is not applicable in the case of analyzing deep beams behavior, as plane sections do not remain plane after the crack of concrete [9]. Considering the structural behavior, the load transfer mechanism of deep beams occurs through concrete struts, with the supports forming the arching effect, and further results in higher shear strength [7,10]. Deep beams serve as load transfer and support elements of offshore based gravity structures [1]. Web openings are often used in such beams to make doors, windows and other facilities, such as ventilation units and air conditioning ducts, accessible, or to accommodate vital facilities [11,12]. Extending such openings because of mechanical demands or changing building functions reduces the shear ability of the element, and thus poses a serious safety risk [11].

In the construction of high-rise buildings, utilities pipes and ducts accommodate essential services such as water, gas and electricity supply, which require a significant number of pipes or tubes [10]. In the past, such tubes or conduits were generally hung under the concrete sheet that was covered by a suspended ceiling, forming a dead room [13]. With the presence of dead space, the building is said to be higher than the required headroom, hence, it increases the construction cost [14]. With the growth of the construction industry, openings in different forms are provided through floor beams for the flow of utility pipes and conduits. It has become a relevant practice in avoiding the problematic issue of headroom induced by the suspension of tubes and conduits [15]. It is thus crucial that these tubes and ducts are passed through an opening in a floor beam, to decrease the headroom and offer a more compact and cost-effective structure. The shape of the openings vary in the form of circular, square or rectangular. The circular opening is usually used in electricity wiring, telephone lines and computer networks, while air-conditioning services use square or rectangular openings [15,16,17]. Such openings in RC beams raised many concerns about the structural functions of the members [17,18]. This makes the simple beam operation more complicated [14,17]. As the cross-sectional sizes of a beam are suddenly changed, the opening angles are subject to elevated levels of stress, which could contribute to broad cracks that are not aesthetically and durably permissible [14,15]. Furthermore, openings on the reinforced concrete beam in web section reduce its rigidity. This can lead to multiple cracking and distortions, severely damaging the resistivity and stability of the beam [19,20]. The reduced area in the total cross-sectional dimension of a beam changes the simple beam behavior to a more complex one [14,21]. To recover the load carrying capacity of the RC deep beam, it is crucial to strengthen the vicinity of such an opening. RC beams comprising openings must, therefore, be appropriately constructed and inspected for strength and stability in order to minimize the losses [22,23].

**Figure 2:**
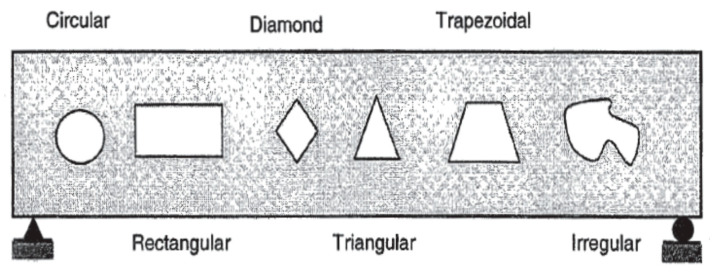
Different forms of opening shapes [32].

The shear behavior is the main cause that could generate disturbances in internal stresses of the deep beam structure [2,6]. In shear behavior, compression grows in one orientation, whereas tension grows in vertical orientation [6]. As the depth of the beam increases, the shear behavior results in sudden failure [10,24]. Due to the brittle nature, crack propagation in larger size deep beams is much higher than in smaller size deep beams [25]. Failure of deep beams occurs due to crushing of concrete in the compression region of nearby supports or directly along the shear crack formation [10,26]. Khaldoun and Khaled [27] studied the deep beam with a span to depth ratio of 2.5 and observed some reserve strength in the post cracking region, resulting in less brittle behavior [6]. Ashour and Morley [28] concluded that the span to depth ratio of the beam has a significant effect on load carrying capacity, due to horizontal and vertical web reinforcement. The effectiveness of horizontal shear reinforcement in deep beams is much higher than that of vertical shear reinforcement [29]. Russo et al. [30] developed an expression that describes the shear strength of deep beam using the strut and tie model. Diagonal struts, longitudinal reinforcement, vertical stirrups and horizontal web reinforcement are the governing factor in the shear strength of the deep beam. Nair et al. [31] studied deep beams with square and circular shaped openings designed on the basis of the strut and tie model using a finite element analysis. On the basis of the experimental result, circular opening beams offered higher shear resistance than beams with square openings. Similarly, Mansur and Tan [15] stated that transverse openings in the deep beam can be of a different shape and size. However, the most common and preferred openings are square and circular [1]. In 1968, Prentza [32] carried out an extensive research on the various shape and size of transverse opening. Prentza considered different forms of openings, including rectangular, circular, square, diamond, triangular, trapezoidal and irregular shape (Figure 2) [1,32].

As per the literature reviewed, there is no clear and precise definition for the size of openings. Various researchers often use the term small and large openings without any differentiation [10]. Mansur and Hasnat [33] considered the openings of circular, square or almost square shaped as small openings. In a similar study, Somes and Corley, in 1974, defined large openings. According to them, circular openings whose diameter exceeds 0.25 times the depth of the deep beam are considered large openings [34]. Further, transverse openings in deep beams develop the complexity to understand its structural behavior [10].

The reinforcement of beams with openings relies in particular on its categories. In the construction industries, openings in deep beams are basically categorized as pre-planned or post planned openings [35]. Factors such as location, size and shape are identified in advance during the construction phase in pre-planned openings [16,36]. Hence, it is advantageous for adequate strengthening and serviceability of beams with openings during the construction practice. This factor improves the strengthening behavior of a deep beam with openings [23,37,38]. Considering the case of post-planned openings, the existing beam element is drilled for openings that affect the structural performance. However, specific guidelines or standards regarding the openings in the structural element are presently unavailable in any of the major codes [15]. During the laying procedure of utility pipes and conduits, problems may occur [16]. The opening positions in the deep beam are provided or re-located by the mechanical and electrical engineers [39]. The structural engineer analyses the identified drilled position so that it may not exist in the critical region [16]. This simplifies the opening positions in the structural element for the arrangement of pipes and conduits that, during the construction phase, may not be regarded [10]. The simplification in the installation of longer pipes and ducts results in enormous time savings, lower manpower and cost effectiveness, particularly in a multistory construction [16]. The construction of these openings largely interrupts the load transfer mechanism that could result in the structural failure [16,40]. On the basis of previous studies, failure in the deep beam is controlled by its shear capacity [40,41].

In order to enhance shear capacity and re-gain the strength of the affected deep beam, it is treated with exterior reinforcement material, i.e., fiber reinforced polymer (FRP) [16]. FRP composites consist of fiber, resin, interface, fillers and additional compounds [42]. Because of the increased deformation module, fiber contributes to the FRP mechanical strength, whereas resin aids in the transfer or distribution of stress from one fiber to another, in order to protect the fiber from environmental and mechanical damage [42]. The fiber-matrix interface is considered to impact the efficiency of FRP composites considerably [43]. Moreover, fillers serve to reduce cost and shrinkage, whereas additives assist in improving the mechanical and physical properties, as well as the workability of composites [26,43]. Fiber reinforced polymer strengthened the deep beam externally, and other such structural members that are affected during drilling process [10]. According to Tuakta (2004) [44], various types of FRP sheets practiced in the industries include carbon fiber reinforced polymer (CFRP), glass fiber reinforced polymer (GFRP), aramid fiber reinforced polymer (AFRP) and basalt fiber reinforced polymer (BFRP). In comparison with other FRPs, glass fiber reinforced polymers are comparatively low-cost fibers. It is the most widely used material in the building sector. However, the primary disadvantages of GFRP include a reduced deformation module, reduced moisture and alkaline resistance, as well as reduced durability owing to stress fracture [45,46,47]. Polymers with a greater static and impact strength behavior are termed AFRP. However, their use is restricted both by decreased durability, as well as UV radiation sensitivity. A further disadvantage of Aramid fiber includes the difficulty towards its cut and process technique [48,49]. On the other hand, BFRP is a very durable material, with elevated temperature resistance and strong tensile strength. Further benefits include elevated acid resistance, superior electro-magnetic characteristics, corrosion resistance, UV and radiation resistance and excellent vibration resistance [50,51]. Lastly, CFRP has a large deformation modulus and greater fatigue resistance, as well as no water absorption [52]. Therefore, CFRP is the most widely used external strengthening material and is widely recommended by industries and researchers, due to its outstanding qualities, i.e., higher tension resistivity, lighter weight, being resistant towards corrosion, bending resistance, the restoration of its overall structural strength and its ease of application in the construction field [17,53,54,55].

On the basis of past studies, various researchers have explored the structural behavior of reinforced concrete deep beams and its strengthening performance using external materials [7,8,9]. However, due to its complex behavior and deformed geometry, limited research is available concerning the structural behavior of openings in deep beams [10,39]. The current of opening in the web of a RC beam contributes to numerous issues in the beam performance, for example, a decrease in the beam stiffness, excessive cracking, excessive deflection and a decrease in the beam strength [56]. Moreover, a sudden alteration in the dimension of cross-section of the beam led to high stress concentration at the corners of opening, which may contribute to cracking that is intolerable from aesthetic and durability perspectives [57]. The abridged stiffness of the deep beam may also give rise to excessive deflection under service load, and cause a significant redistribution of internal forces and moments in a RC deep beam. Fibrous material, such as CFRP sheets, can increase the structural integrity of the RC deep beams [58]. These fibers of CFRP laminations are generally uniformly distributed and randomly oriented. CFRP sheets can increase structural strength, decrease steel reinforcement requirements, improve ductility, reduce crack width, control crack width tightly, improve durability and enhance freeze-thaw resistance. Due to the uniform distribution of CFRP fibers, it behaves as a three-dimensional reinforcement. Additionally, the available literature considering the strengthening behavior of deep beams using CFRP is also quite limited [59,60]. Therefore, this research investigates the structural performance of un-strengthened and strengthened behavior of reinforced concrete deep beams with openings strengthened with CFRP layers in terms of cracking pattern, mode of failures, load-deflection behavior, stress concentration factor and strengthening response.

## 2. Experimental Program

### 2.1. Specimen Details

**Figure 3:**
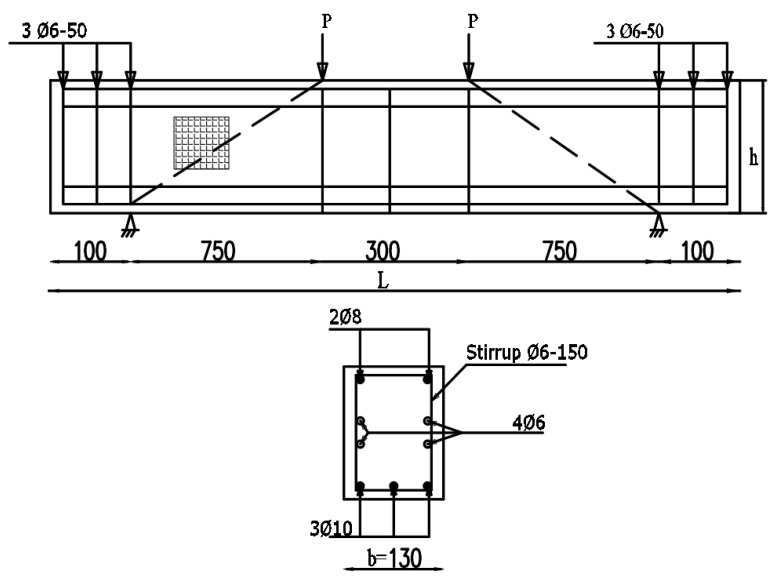
Arrangement of the reinforcement in the tested deep beams (All units in mm).

This study included a control RC deep beam and nine RC deep beams with an opening in the shear zone. All the 10 deep beams had a 130 mm (b) × 500 mm (h) cross-section and were 2000 mm long (L), as shown in Figure 3. Considering the reinforcement, two of the 8 mm diameter plain steel bars and three of the 10 mm diameters deform bars were used as a compression reinforcement and tension reinforcement respectively. Other than that, 4 plain steel bars with a 6 mm diameter were placed in the middle of the reinforcement. A 6 mm stirrup with a 150 mm spacing was used with 15 mm of concrete cover.

A total of 9 RC deep beams with openings were separated into 3 groups (Table 1), based on the size of openings including 150 mm (Group B1), 200 mm (Group B2) and 250 mm (Group B3). Each group consists of 3 beams (a–c) with various numbers of CFRP layers; one (a), two (b) and three (c) layers.

In order to verify the usability of the above-mentioned models in predicting of the shear capacity of RC beam with web opening strengthened by CFRP (single and multiple layers); analytical analysis is carried out in Section 4.

### 2.2. Specimens Preparation

After the reinforcement was prepared, a Kyowa strain gage type KFG-5-120-C1-11 (Kyowa Electronic (Shang Hai) Trading Co., Ltd, Shang Hai, China) was attached at the bottom of the lower deform bar to measure the strain experienced by the deep beam under a four-point bending load. The gage leads were connected to the gage terminal by soldering, to ensure that strain encountered by the deep beam was transmitted in the form of an electrical resistance that can be measured. A well functioned strain gage was given a voltmeter reading of 120.2 ± 0.2 Ω.

The lower deformed bar with 10 mm diameter was ground to make a flat surface before the strain gage, and the terminal was attached to it. Acetone was used to clean the surface from any dirt and stains for a better adhesive surface. HA3000 RTV Silicone Rubber was used to protect the sensitive layer of strain gage against humidity. The strain gage needed to be wrapped properly to avoid water from the concrete paste penetrate and reach the surface of the strain gage, which hence affects the result.

In this experimental study, all the deep beam specimens were cast using the ready-mixed concrete of grade 25 MPa, obtained from the LTH Cement Plant (Perak, Malaysia), and tested for compression behavior at 28 days duration. Wooden formworks were used as a mold for the beams. Grease needed to be applied to the formwork to ease the process of the striking of formwork that was done after 3 days from the casting time, followed by curing of the deep for 28 days to get the optimum strength.

The type of CFRP laminates used in this experiment was the SikaWrap Hex 230C (Sika Kimia Sdn. Bhd., Negeri Sembilan, Malaysia) with 100 mm width on each side. Before applying the CFRP, the surface of the concrete was ground and cleaned using acetone for better adhesion. CFRP was attached around the opening for 1 layer and up to 3 layers for each beam, with an opening following the details in Table 1. Sikadur 330 (Sika Kimia Sdn. Bhd., Negeri Sembilan, Malaysia) a mixture of epoxy resin and adhesive, was used to bond the CFRP to the surface of the concrete. The epoxy was applied twice, before and after attaching the CFRP, to make the CFRP sandwich with the epoxy and ensure that it was strongly bonded to the concrete. Safety gloves and face masks had to be worn throughout the process.

### 2.3. Experimental Set-Up

**Figure 4:**
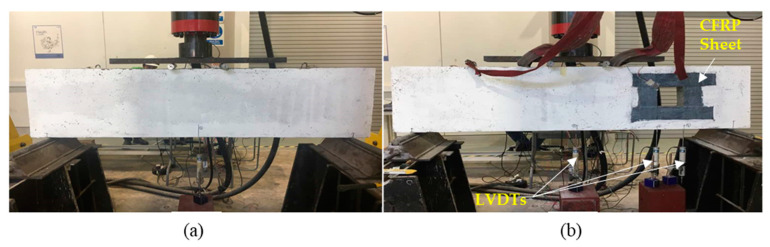
Equipment and testing setup: (**a**) control beam; and (**b**) deep beam with opening.

**Figure 5:**
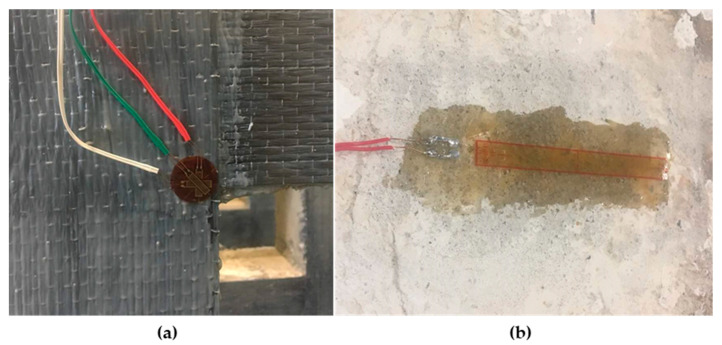
Arrangement of: (**a**) rosette strain gauge; and (**b**) uniaxial strain gage.

All beams were tested until failure under four-point bending loads with a loading rate of 1 kN/s, using a self-straining loading frame. The load was transferred symmetrically through two loading points, placed at 850 mm away from the edge of the beam. During the test, the beam specimen was placed on two supports, with 100 mm distance from each edge of the beam. In order to observe the beam deflection, linear variable displacement transducers (LVDTs) were positioned at the center of the beam bottom soffit and at the end of the openings (Figure 4). A CFRP sheet was attached around the square opening, located 250 mm away from the point load. The size of the opening was 150, 200 and 250 mm. The CFRP sheet was fully wrapped around the opening, at both the tension and compression zones, to investigate the effective number of CFRP layers used to intercept the diagonal cracking when the load is applied. Rosette strain gauge and uniaxial strain gage were attached at the corner of CFRP and on top of the beam, respectively (Figure 5), by using super glue, after cleaning the surface with acetone. Araldite Epoxy Adhesive glue (Huntsman Advanced Materials, Basel, Switzerland) was used to make a flat surface on top of the concrete, before attaching the uniaxial strain gage. Rosette strain gauge was used to measure the strain of CFRP along 3 different directions, while uniaxial strain gauge was used to measure the strain of concrete in one direction only.

## 3. Results and Discussions

### 3.1. Failure Modes and Cracking Patterns

#### 3.1.1. Control Beam (CB)

**Figure 6:**
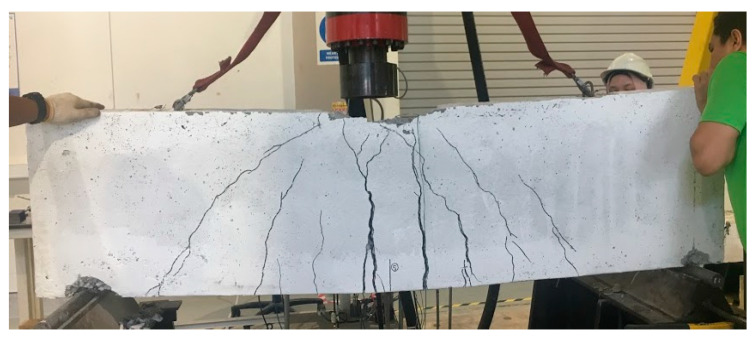
The crack patterns of control beam at failure.

When the load was placed on the deep beam, the first small crack sections appeared at the center of the span which is the tension region and it becomes more visible as the load is applied continuously with an increment of 1 kN/s. The cracks changed into significant cracks and propagated up to the neutral axis (NA). Diagonal cracks were observed near the support and penetrate to the loading point until concrete crushing occurred at both point loads and supports. Both flexural and shear cracks were noticed within the span, until the control beam experienced a sudden failure due to a wider crack width. The crack patterns for the control beam at failure are shown in Figure 6.

#### 3.1.2. Deep Beams with Web Opening

The beams with opening was divided into three groups. The first group considered the deep beam with web opening of 150 mm. For these beams, the crack patterns started with a minor crack line, until the cracks were obvious at the tension zone up to the NA area that was free from the CFRP sheet. The early cracks were marked to detect the development of the crack. The cracks were then propagated to the strengthened opening until failure.

**Figure 7:**
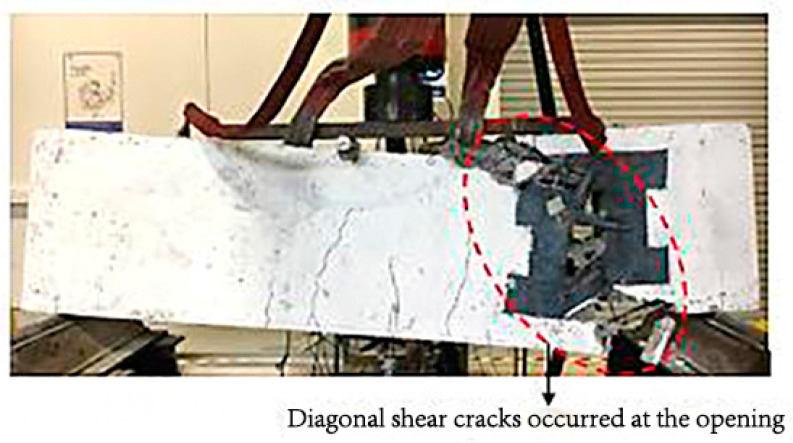
Failure mode and cracking pattern of the deep beam, B1a, at failure.

For deep beam B1a, having 1 layer of CFRP around the opening indicates that the sudden diagonal shear cracks occurred until the concrete and CFRP were torn apart, while the bottom rebars were exposed and bent downwards, as shown in Figure 7. The provision of the opening has disturbed the natural load path; hence, CFRP was used to divert the load away from the opening. However, one layer of CFRP is not enough to withstand the stress concentration from the load, resulting in sudden failure and occurred at the opening ends of the deep beam.

**Figure 8:**
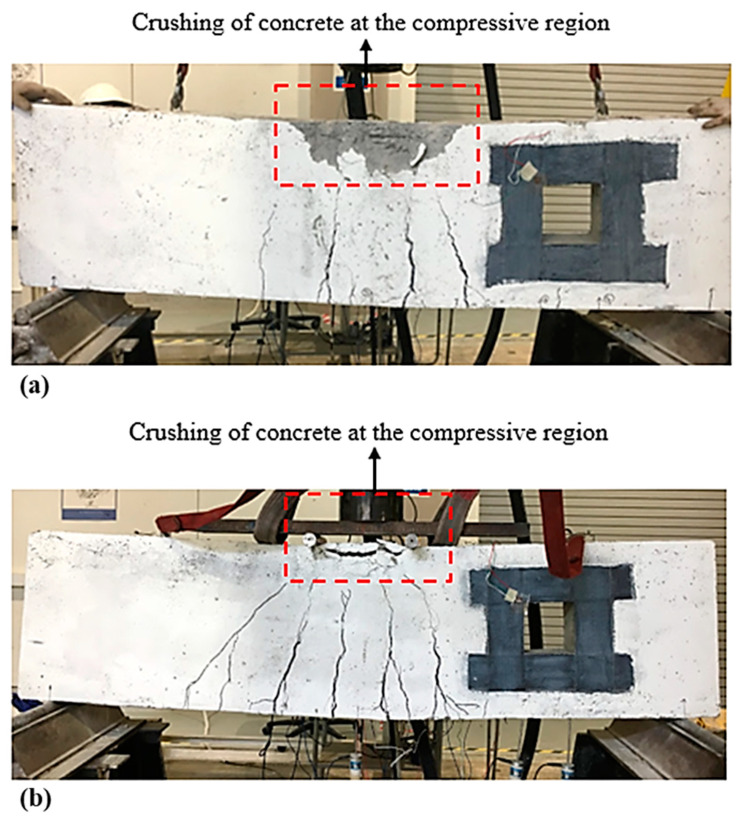
Failure mode and cracking pattern of the deep beam (**a**) B1b and (**b**) B1c.

For the deep beam, B1b and B1c, having two and three layers of CFRP sheet, correspondingly, indicate that the same initial cracks occurred. Minor flexural cracks appeared in the tension region that is eventually wider in width as the load increased. There were no diagonal shear cracks observed at the strengthened opening for both deep beams, however, the crack lines appeared at the center of the deep beam soffit, until it reached its ultimate failure. The crushing of concrete in the compressive region was obvious due to the localized shear stress. It was observed that both deep beams are placed under flexural failure. Figure 8a,b illustrates crack patterns within the beam cross-section at failure.

**Figure 9:**
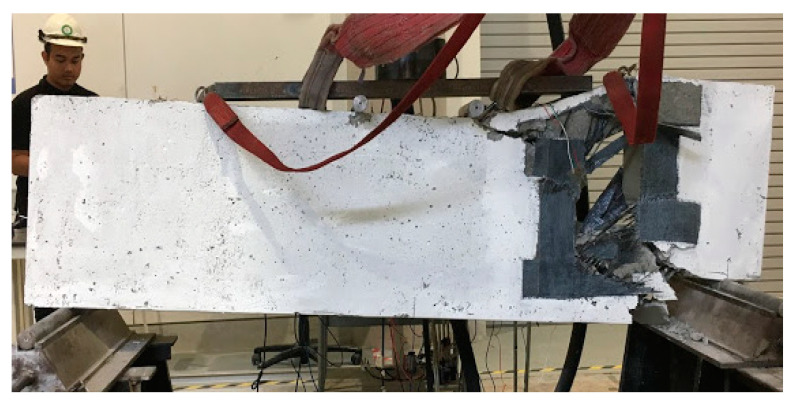
Failure mode and cracking pattern of deep beam B2a.

For the Group 2 deep beams, having one CFRP layer, B2a, the deep beam failed suddenly, as the load was applied with no minor crack lines at the tension zone (Figure 9). This is due to a large square opening that caused a large disturbance to the natural load path. One coating of CFRP was insufficient to divert loading route and resulted in the failure of the deep beam (shear failure). This failure was occurred in specimen B2a (Figure 9) where the CFRP and concrete broke apart. Some parts of the CFRP detached from the concrete, and the upper and lower steel bars were exposed due to the splitting of concrete. Figure 8 shows the shear failure at the strengthened opening with a low ultimate strength capacity.

**Figure 10:**
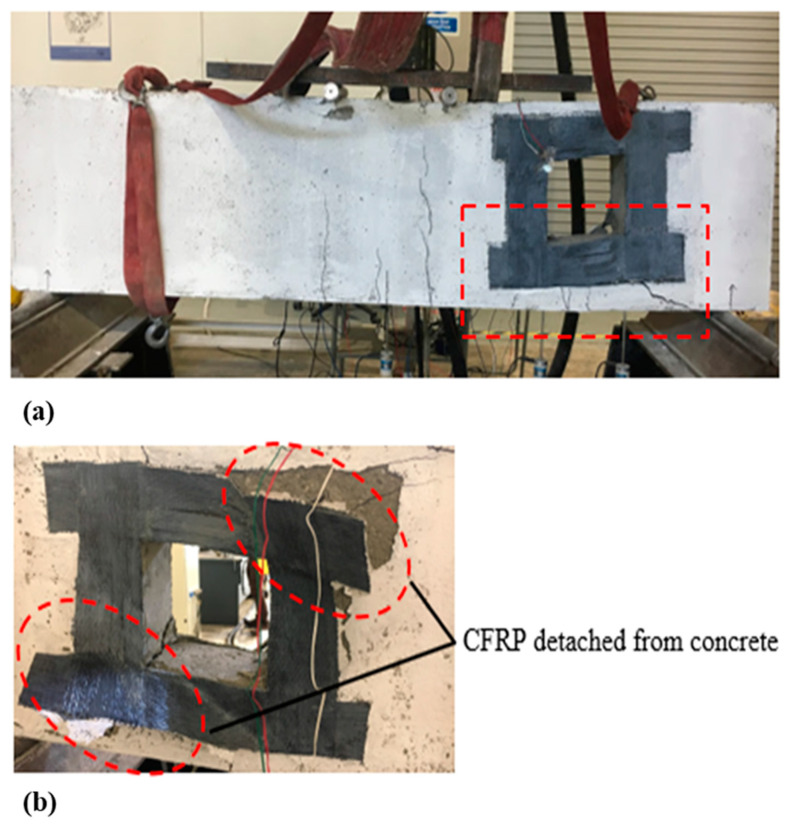
(**a**) Failure mode and cracking pattern of deep beam B2b; and (**b**) detachment and peeling of the carbon fiber reinforced polymer (CFRP) sheet from the concrete.

For deep beam B2b, having two layers of CFRP sheets, the initial minor cracks were observed at the soffit of the beam up to the natural axis (NA). As the load increased, diagonal cracks appeared at the opening, with CFRP ruptured at one side and detachment from the concrete at the other side, due to the high rigidity of CFRP sheet. There was no crushing of concrete in the compression and tension region. Figure 10a shows that deep beam B2b experienced shear failure when it reached the ultimate load capacity. Figure 10b represents the detachment and peeling of CFRP sheet from concrete.

**Figure 11:**
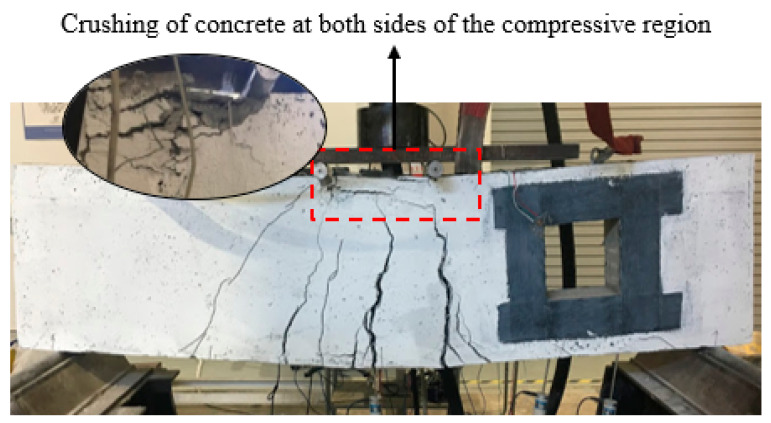
Failure mode and cracking pattern of deep beam B2c.

A deep beam with three layers of CFRP sheets, B2c showed initial flexural cracks motivated to be changed into significant cracks up to two-point loads (Figure 11). Shear cracking started to occur at edges of the deep beam with no openings. No damage to the opening and CFRP sheet was observed.

**Figure 12:**
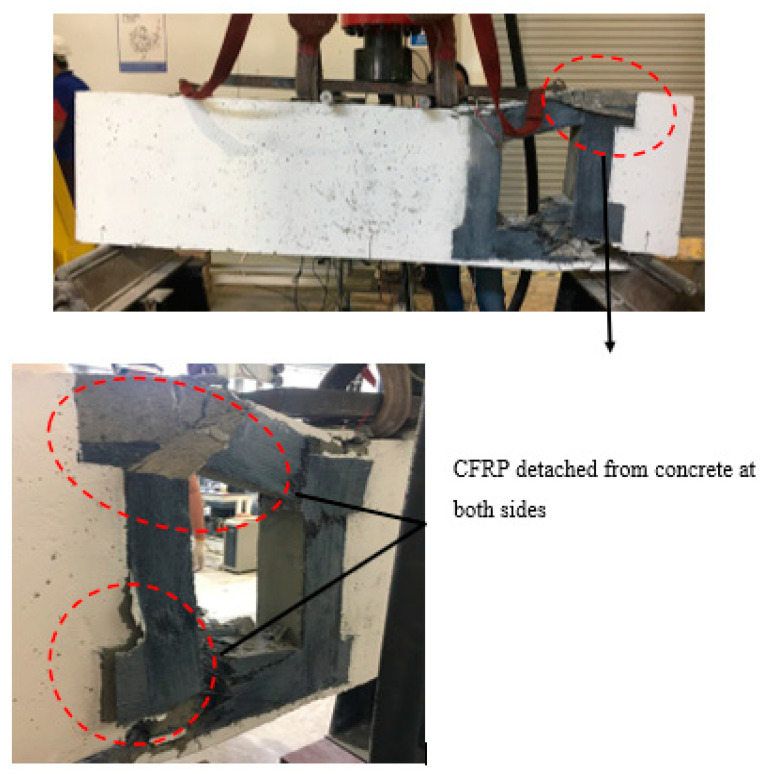
Failure mode and cracking pattern of deep beam B3a at failure.

**Figure 13:**
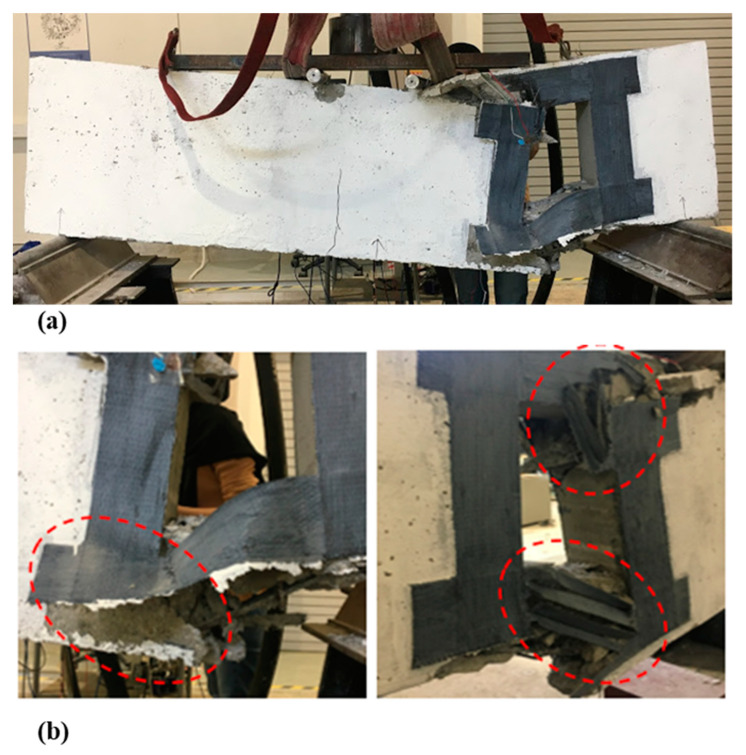
(**a**) Failure mode and cracking pattern of deep beam B3b; and (**b**) CFRP detached from the concrete and tore into two for deep beam B3b.

**Figure 14:**
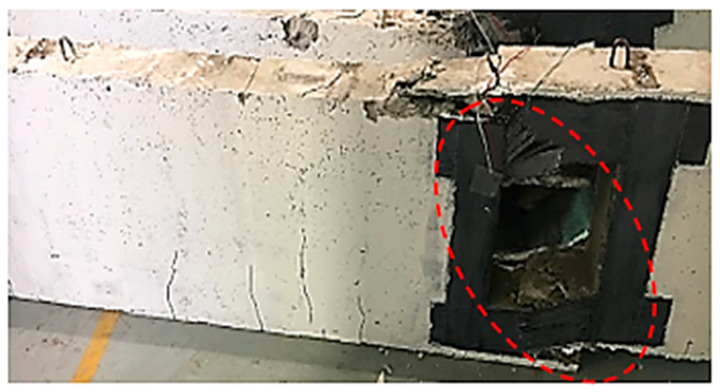
Failure mode and cracking pattern of deep beam B3c.

For the largest size of the opening (Group 3), (250 mm × 250 mm), all three deep beams with one, two and three layers of CFRP failed in shear with CFRP, and the concrete tore into two. Initially, only a few flexural crack lines appeared at the tension region until sudden shear failure occurred. This shows that CFRP with 100 mm width is not enough to strengthen the opening externally, regardless the number of layers of CFRP attached around the opening. With a large size of opening, the reduction of strength capacity increased and resulted in sudden shear failure. Figure 12, Figure 13 and Figure 14 illustrate that the shear failure occurred at the opening of deep beam B3a, B3b and B3c, respectively.

**Figure 15:**
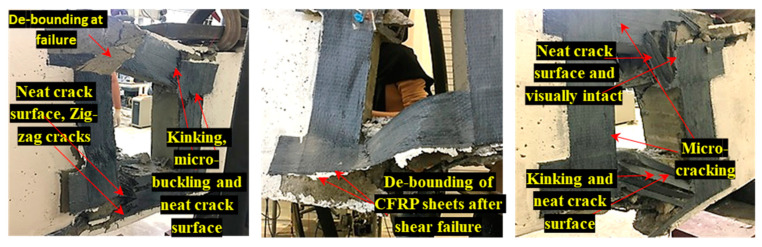
Typical post-mortem photos after the static four-point bending tests of the deep beams with opening.

Figure 15 shows the typical failure photos of the deep beams after the static four-point bending tests at a different opening size and various layers of CFRP. It was revealed that the post-mortem photos show major cracks that are localized in the mid-section perpendicular to the fiber direction, and caused de-bounding of CFRP sheets at failure load. In addition, the upper half crack surface caused by compression shows a neat fracture surface at failure of shear load, while the lower half crack surface caused by tension shows a rough zig-zag fracture surface. The upper layers stay visually intact, and the damage is delocalized by many secondary cracks parallel to the fiber direction. At a failure mode, the transition of cracks from the upper layer of micro-buckling to the bottom layer tensile breakage is revealed and led to the formation of extensive micro-cracks when the load is reached at the ultimate point of fracture. On the other side, interlaminar shear failure was not observed, and the only noticed failure was de-bounding, with peeling of the concrete cover between the CFRP sheets and the concrete surface at the failure load.

### 3.2. Load-Deflection Behaviour

**Figure 16:**
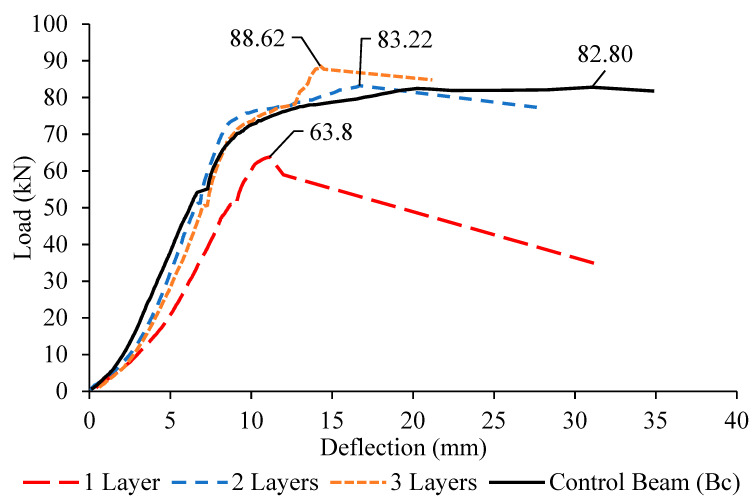
Load–deflection curves for Group 1 (B1a–B1c) deep beams and control beam.

**Figure 17:**
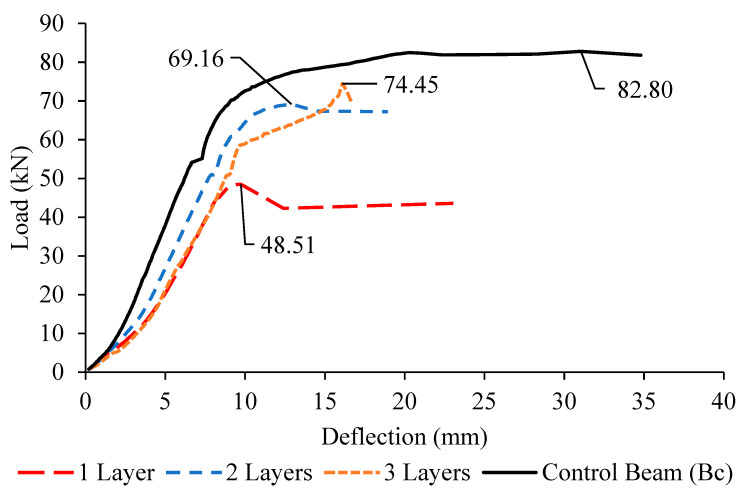
Load–deflection curves for Group 2 (B2a–B2c) deep beams and control beam.

**Figure 18:**
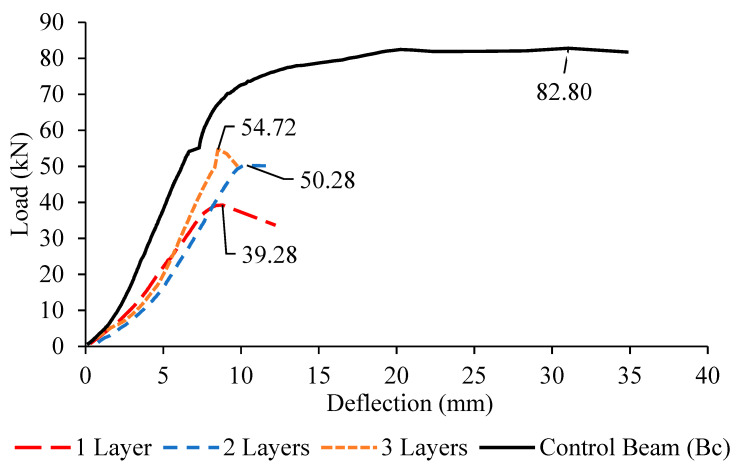
Load–deflection curves for Group 3 (B3a–B3c) deep beams and control beam.

The load-deflection behavior was observed and recorded for all beams. The curves were shown in Figure 16, Figure 17 and Figure 18. From the curves plotted with comparison to the control deep beam, the highest deflection exhibited by the control deep beam with 34.8 mm, followed by the deep beam with one layer, two layers and three layers of CFRP. This is because of the presence of the external strengthening system CFRP, which enhanced the concrete stiffness and rigidity, resulting in the decreasing of mid-span deflection.

By referring to the graphs above, it is proven that, by attaching the CFRP sheet around the opening, the stiffness of the concrete increases from about 10% to 30%. Taking the Group 1 deep beams as an example, with the presence of one layer of CFRP, the deflection is 31.62 mm, while the deflection for deep beams with two and three layers of CFRP is 28.01 mm and 21.09 mm, respectively. This result proves that the CFRP sheet enhanced the rigidity of the concrete; hence, it lowered the bending at the mid-span. As the number of CFRP layers increases, the mid-span deflection decreases with not much difference.

However, in comparison to the number of CFRP layers, the smallest opening, 150 mm exhibits the largest deflection, followed by the opening sizes of 200 and 250 mm, due to significant shear strength reduction to about 10% to 30% of the ultimate load. The deep beam with an opening of 250 mm fails in shear earlier than the ones with the opening sizes of 200 and 150 mm, with a lower ultimate load capacity, giving not enough time for the deflection to occur.

### 3.3. Flexural Strength

**Figure 19:**
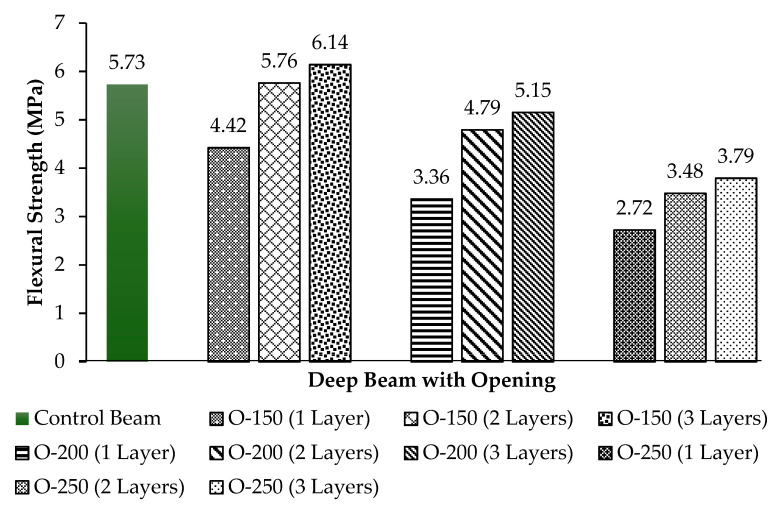
Flexural strength results of control deep beam and 3 groups of deep beams with opening.

Flexural strength is measured by Equation (1) to check for the tensile strength of the concrete, and resists the failure in bending from occurring. The chart shown in Figure 19 shows the comparison of flexural strength for all deep beams.

(1) $$\sigma_{\mathrm{fs}}=\frac{\mathrm{Pu}\;\left(\frac{a}{2}\right)}{\frac{1}{6}{\mathrm{bh}}^{2}}$$

where; $\sigma_{\mathrm{fs}}$= flexural strength (MPa), $P_{u}$ = ultimate load (kN), a = shear span of the beam (mm), b = width of deep beam (mm), h = height of deep beam (mm).

The highest flexural strength exhibited by the deep beam with the opening size of 150 mm and strengthened with three layers of CFRP (Figure 18). This shows that even the existence of the opening had resulted in a shear strength reduction, and that CFRP helps in reserving the flexural strength in a way that can carter the load applied to it. In other words, it helps increase the flexural strength and shear strength capacity. However, with an increase of the opening size, the flexural strength decreases due to low shear strength capacity.

### 3.4. Stress Concentration Factor, k

From the reading of the rosette strain gage attached at the corner of the opening, one analysis of the stress concentration factor (SCF), k, is performed by using the following equations:

(i) stress concentration factor (SCF)

(2) $$k=\sigma_{1}.\sigma_{nom}$$

where $\sigma_{nom}$ is the nominal stress $\sigma_{nom}=0.8P_{u}A_{cs}$; $A_{cs}$ = vertical shear plane and $\sigma_{1}$ is the principal stress

(3) $$\sigma_{1}=0.5\left(\sigma_{x}+\sigma_{y}\right)+{\left[{\left(\frac{\sigma_{x}-\sigma_{y}}{2}\right)}^{2}+{\left(\tau xy\right)}^{2}\right]}^{0.5}$$

**Figure 20:**
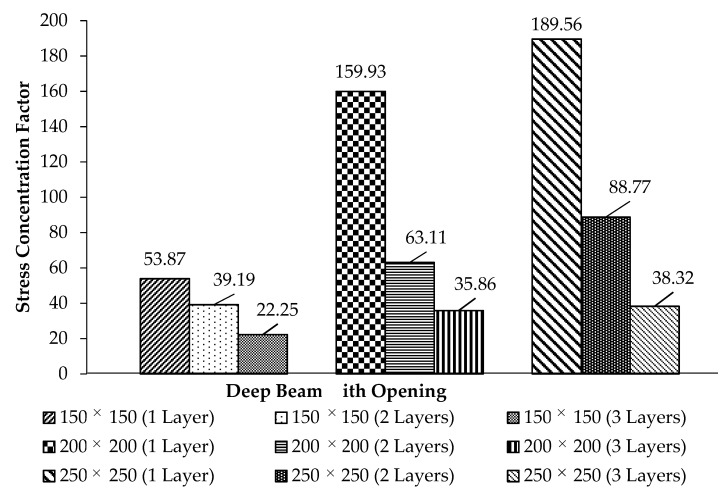
Flexural stress concentration factor (k).

From Figure 20, the largest size of the opening, 250 mm with one layer of CFRP, indicated the highest value of SCF. This shows that the large size of opening experienced high stress in the beam cross-section, and that having one layer of CFRP is insufficient to strengthen the opening and resulted in early failure with low shear strength capacity. By increasing the number of CFRP layers, the stress concentration factor is reduced by 30% to 60%. It can be concluded that CFRP helps in minimizing the risk of stress failure.

## 4. Analytical Analysis

A comparison of the experimental shear capacities of the tested beams having a CFRP strengthened opening with three existing theoretical models. It can be observed that the experimental results were in agreement with the Eurocode 2 model. Although the Khalifa Model and the CSA Standard model [61] overestimated the shear force due to the safety factors included in those models.

To understand the behavior of openings in the reinforced concrete deep beams various researchers have adopted experimental and numerical techniques [16,23,62].

### 4.1. Triantafillou Model (Eurocode 2)

Triantafillou (1998) [63] researched FRP strengthening contribution to RC beam shear ability. His research showed an equation that refers to the strain of FRP to the RC beam shear breakdown (effective strain) of directly connected bands or plates, to their axial rigidity. The FRP response to shear strength is based on Eurocode 2 and transcribed as per Equation (4) [63]:

(4) $$V_{f}=0.9\frac{\varepsilon_{\mathrm{fke}}}{\gamma_{f}}E_{f}\rho_{f}b_{w}d_{t}\left(\mathrm{Sin}\beta +\mathrm{Cos}\beta \right)\;$$

where; γ_f_ = partial CFRP safety factor, i.e., 1.15, $E_{f}\;$= CFRP elastic modulus in the main fiber direction, i.e., 230 GPa, β = angle between the primary fiber position and the opening chord longitudinal axis, $\varrho_{f}$ = strengthening proportion FRP = $2t/b_{w}$, t = thickness of FRP laminates, $d_{t}$ = top cord effective depth, and $\varepsilon_{\mathrm{fke}}$ = effective FRP strain characteristic value, it mainly depends on the FRP “development” length, defined as that necessary to reach FRP tensile fracture before debonding.

### 4.2. Khalifa Model

Khalifa et al. (1998) [64] modified Equation (4) slightly in order to calculate the RC beam shearing capacity based on predicted failure types including Type I: concrete cracking shear; Type II: breakage of FRP layers, and,;Type III: FRP layer bond failure bonded to concrete. In particular, a reduction factor (R) was suggested to be added to Equation (4), as presented in Equation (5).

(5) $$V_{f}=0.9\varrho_{f}b_{\mathrm{wt}}d_{t}{\mathrm{Rf}}_{\mathrm{fu}}\;\left(\mathrm{Sin}\beta +\mathrm{Cos}\beta \right)\;$$

where; R = FRP stress reduction factor; $f_{\mathrm{fu}}$ = FRP layer ultimate tensile stress in the primary fiber direction.

In addition, R must be considered as the smallest of the three following values (Equations (6)–(9)).

For Failure Type I:

(6) $$R\;=\frac{\varepsilon_{\mathrm{fe}}}{\varepsilon_{\mathrm{fu}}}$$

Since CFRP is linearly elastic till failure, the effective stress can be calculated by Equation (7).

(7) $$R\;=\frac{f_{\mathrm{fe}}}{\mathrm{fu}}$$

For Failure Type II:

(8) $$R=0.778-1.218\rho_{f}+0.5622{\left(E_{f}\rho_{f}\right)}^{2}$$

For Failure Type III:

(9) R=0.0042(fc)23  Wfe(Ef. tf)0.58εfu df

where; $\rho_{f}=$ strengthening proportion, R = stress reduction factor, $f_{\mathrm{fu}}$ = ultimate tensile stress, $\varepsilon_{\mathrm{fe}}$ = effective strain, $\varepsilon_{\mathrm{fu}}$ = ultimate tensile strain, $f_{\mathrm{fe}}$ = effective tensile stress, $E_{f}\;$= elastic modulus, $w_{\mathrm{fe}}$ = effective length, and $t_{f}$ = Layer thickness.

### 4.3. Canadian Standards Association (CSA) Model

The Canadian Standards Association (CSA S806) [65] has proposed a simpler strategy to evaluate effective FRP stress as shown in Equation (10):

(10) $${\;V}_{\mathrm{ft}}=0.9\rho_{f}b_{\mathrm{wt}}d_{t}f_{\mathrm{fe}}\;\left(\mathrm{Sin}\beta +\mathrm{Cos}\beta \right)$$

where;

$$f_{\mathrm{fe}\;}=E_{f}\varepsilon_{\mathrm{fe}}$$

In which the value of effective strain $\left(\varepsilon_{\mathrm{fe}}\right)$ is considered to be 0.004. Hence the FRP stress is given by (${\mathrm{Rf}}_{u}$) from Equation (6) has been replaced by $f_{\mathrm{fe}}$. To evaluate the outcome of CFRP reinforced beams without a web opening, the above models were developed.

For these models to be adapted to study the behavior of RC strengthened CFRP beams with web openings, the shear resistance of FRP with the present openings in the shear area should be modified to a small extent. The effective depth (d) is therefore substituted in each equation by the net depth (d-d_o_) [62].

A comparison of the experimental shear capacities of the tested beams having a CFRP strengthened opening with the three models results were shown in Table 2. It can be observed that the experimental results were in agreement with Eurocode 2 model. Although the Khalifa Model and the CSA Standard model overestimated the shear force due to the safety factors included in those models. Therefore, the slightly modified Eurocode model derived for the solid beam (without opening) can be used in predicting the shear capacity with reasonable accuracy.

## 5. Conclusions

This experimental study aimed to examine the structural behavior of the deep beam with a web opening strengthened using CFRP. The effect on the deep beam behavior includes cracking pattern, mode of failure and load-deflection, perceived through the size of openings and number of CFRP layers considered during sample preparation. On the basis of the experimental result obtained, it is proven that the external strengthening method, by using CFRP, significantly helped to upgrade the strength of the deep beam, observing that the load path is commonly disturbed by the opening. For opening size with 150 mm, one layer of CFRP was not adequate to divert the load path, resulting in shear failure. However, with the application of two and three layers of CFRP, the beams failed in flexural. The single and double layers of CFRP around the opening size of 200 mm were not sufficient to intercept the load path, resulting in shear failure. However, the triple layer is remarkably sufficient in enhancing the shear strength gain, and caused the deep beam to fail in flexural. For opening size with 250 mm, even three layers of CFRP were not adequate to sustain the shear strength, due to high shear strength reduction. Hence, all beams failed in shear.

In terms of flexural strength, it can be concluded that increasing the number of CFRP layer can significantly lead to a reduction in the deflection at mid-span, due to an increase of member stiffness from about 10% to 40%. Moreover, increasing the size of the opening can decrease the deflection at mid-span, as a consequence of a high shear strength reduction of up to 30%. Hence, the larger the size of the opening, the lower the load carrying ability. The ultimate load carrying ability of the deep beam with the largest opening size of 250 mm and a single layer of CFRP was found to be 39.28 kN, whereas, compared to the deep beam having an opening size of 150 mm and a single CFRP layer, the ultimate load carrying ability found to be 63.8 kN. Furthermore, increasing the number of CFRP layers resulted in an increase in the load carrying capacity. The CFRP enhances the shear strength gain from about 10% to 40%. Consequently, the most effective number of CFRP layers for the deep beam with opening size with 150 mm was a double layer, while for the opening size with 200 mm it was a triple layer. However, it can be concluded that this experimental study is a valuable contribution for construction personnel such as structural engineers, as it provides clear information for shaping openings within existing beams.

It also has been found that using the modified theoretical model of Eurocode 2 will lead to a reasonable prediction of the shear capacity of RC deep beams with openings strengthened with single or multiple layers of CFRP for safe design purposes.

Structural behavior of strengthened deep beam with varies shapes, sizes and locations of the opening should be considered for further study for both numerical and experimental analysis. Moreover, the experimental study can also be widened into the study of the effects of other strengthening materials: AFRP & GFRP on the deep beams with an opening. Consequently, the current study does not include a control deep beam for each size of opening with no attachment of CFRP. Therefore, for future research, the structural behavior of a control unstrengthened deep beam with a web opening shall be investigated to set as a benchmark for each size of the opening.

## Figures and Tables

**Table 1:** Beam specimen details.

Beam Sample	Size of Openings (mm)		Numbers of CFRP Layers
	Width	Height	
Control Beam (Bc)	0	0	0
B1a	150	150	1
B1b	150	150	2
B1c	150	150	3
B2a	200	200	1
B2b	200	200	2
B2c	200	200	3
B3a	250	250	1
B3b	250	250	2
B3c	250	250	3

**Table 2:** Comparison of theoretical and experimental shear capacity of tested beams.

Beam Sample	$V_{c}\;(\mathrm{kN})$	Eurocode 2 Model		Khalifa Model		CSA S806		$V_{exp}\;(\mathrm{kN})$
		$V_{f}$	$V_{r}$	$V_{f}$	$V_{r}$	$V_{f}$	$V_{r}$	
B1a	21.79	26.17	47.96	59.17	80.96	34.75	56.54	44.52
B1b	21.79	22.44	44.23	59.17	80.96	34.75	56.54	41.12
B1c	18.57	20.96	39.53	45.63	67.42	31.25	53.04	34.22
B2a	21.79	30.34	52.13	48.69	69.39	69.75	90.46	53.24
B2b	21.79	30.34	52.13	48.69	69.39	69.75	90.46	53.24
B2c	18.57	27.51	46.08	47.30	68.01	56.62	77.33	40.23
B3a	21.79	37.92	59.71	131.66	147.11	122.03	137.48	44.18
B3b	21.79	37.92	59.71	131.66	147.11	122.03	137.48	44.18
B3c	18.57	30.16	48.73	110.05	125.49	110.25	125.70	46.19
